# Sensitivity of wild-type and rifampicin-resistant O157 and non-O157 Shiga toxin-producing *Escherichia coli* to elevated hydrostatic pressure and lactic acid in ground meat and meat homogenate

**DOI:** 10.1371/journal.pone.0246735

**Published:** 2021-02-18

**Authors:** Abimbola Allison, Aliyar Cyrus Fouladkhah

**Affiliations:** Public Health Microbiology Laboratory, Tennessee State University, Nashville, Tennessee, United States of America; University of Connecticut, UNITED STATES

## Abstract

Various serogroups of Shiga toxin-producing *Escherichia coli* have been epidemiologically associated with foodborne disease episodes in the United States and around the globe, with *E*. *coli* O157: H7 as the dominant serogroup of public health concern. Serogroups other than O157 are currently associated with about 60% of Shiga toxin-producing *E*. *coli* related foodborne illness episodes. Current study evaluated sensitivity of the O157 and epidemiologically important non-O157 serogroups of the pathogen to elevated hydrostatic pressure and 1% lactic acid. Pressure intensity of 250 to 650 MPa were applied for 0 to 7 min for inactivation of strain mixtures of wild-type and rifampicin-resistant *E*. *coli* O157, as well as O26, O45, O103, O111, O121, and O145 serogroups and ATCC^®^ 43895^™^ strain in ground meat and 10% meat homogenate. *E*. *coli* O157 were reduced (*p* < 0.05) from 6.86 ± 0.2 to 4.56 ± 0.1 log CFU/g when exposed to pressure of 650 MPa for 7 min. Corresponding reductions (*p* < 0.05) for non-O157 *E*. *coli* were from 6.98 ± 0.3 to 4.72 ± 0.1. The *D-*values at 650 MPa were 3.71 and 3.47 min for O157 and non-O157 serogroups, respectively. Presence of 1% lactic acid to a great extent augmented (*p* < 0.05) decontamination efficacy of the treatment in meat homogenate resulting in up to 5.6 and 6.0 log CFU/mL reductions for O157 and non-O157 serogroups, respectively. Among the tested serogroups, the wild-type and rifampicin-resistant phenotypes exhibited (*p* ≥ 0.05) comparable pressure sensitivity. Thus, these two phenotypes could be used interchangeably in validation studies. Our results also illustrate that, application of elevated hydrostatic pressure could be utilized for assuring safety of ground and non-intact meat products against various serogroups of Shiga toxin-producing *E*. *coli*. Addition of 1% lactic acid additionally provided industrially appreciable augmentation in efficacy of the pressure-based treatments.

## Introduction

While vast majority of *Escherichia coli* serovars are commensal microorganisms and do not pose any health risk, a small subgroup of this bacterium could cause intestinal and extraintestinal infections in humans. Among pathogenic *E*. *coli*, there were historically six well-defined pathotypes [1]. An outbreak of *E*. *coli* O104: H4 that simultaneously exhibited characteristics of two previously characterized pathotypes, and advancements in molecular subtyping had however blurred the boundaries among these six categories of pathogenic *E*. *coli* [2, 3], making nomenclature of pathogenic *E*. *coli* a complex and evolving matter. Vast majority of foodborne burden associated with this pathogen is associated with Enterohaemorrhagic *E*. *coli* (EHEC) pathotype. Many of the serogroups belonging to this pathotype are capable of encoding/excreting Shiga toxins and causing hemorrhagic colitis. Additionally, in high risk cases, they could cause a potentially fatal kidney complication in form of hemolytic uremic syndrome [1].

As the most epidemiologically significant serogroup of EHEC, *E*. *coli* O157: H7 had been the causative agent of 1992–1993 hemorrhagic colitis outbreaks of hamburgers in Pacific Northwest of the United States [4]. Since then, the pathogen had been an important part of regulatory affairs and public health risk assessment associated with production of primary and further processed meat products. Specifically, this pathogen became the first microbial agent recognized as an “adulterant” in raw non-intact meat and beef manufacturing trimmings. Emergence of this pathogen in food chain was one of the main driving forces of new and risk-based food policies and regulations in the United States and around the globe [5]. There are more than 400 non-O157 serogroups belonging to EHEC that could additionally cause health complications in human and are associated with the food chain. Among these, six serogroups commonly known as the “big six,” are the remaining most epidemiologically significant members of EHEC in the United States. In descending order O26, O111, O103, O121, O45, and O145 serogroups are collectively the causative agents of >80% of non-O157 Shiga toxin-producing *E*. *coli* human isolates, based on active surveillance data of the U.S. Centers for Disease Control and Prevention [6].

In recent years, these six serogroups had also received important regulatory status in meat industry, similar to *E*. *coli* O157: H7, and since June, 2012 the Food Safety Inspection Service of the United States Department of Agriculture had initiated a testing program for these six serogroups in raw beef manufacturing trimmings of domestic and imported products [7, 8]. Since *E*. *coli* O157:H7 had received this regulatory status many years earlier than the non-O157 serogroups, understandably, vast majority of validation studies in microbiology literature are conducted for decontamination of O157 serogroup. Thus, validation studies comparing both O157 and non-O157 serogroups could be of great importance for stakeholders and practitioners [9–11]. It is noteworthy that based on active surveillance data of the U.S. Centers for Disease Control and Prevention, O157 and non-O157 Shiga toxin-producing *E*. *coli* are responsible for 63,153 and 112,752 episodes of illness in a typical year with a corresponding 46.2% and 12.8% hospitalization rate and 0.5% and 0.3% death rate, respectively [12]. Burden of the pathogen is similar in Europe, based on epidemiological data of European Centre for Disease Control and Prevention. The public health burden associated with Shiga toxin-producing *E*. *coli* had recently increased by about 40% in European Union, evidenced by comparing the epidemiological data from 2014 to 2017 to those obtained in 2018 [13].

Application of elevated hydrostatic pressure for elimination of bacterial pathogens had been proposed theoretically several decades ago. Nevertheless, due to advancements in engineering of the processing units, only in the recent years application of high-pressure processing is gaining increasing importance and momentum in various sectors of food manufacturing [3, 14–16]. The technology exposes the product in final packaging to elevated hydrostatic pressure of 100 to 650 MPa, typically for less than 10 min for achieving microbiological safety via physical elimination of pathogens [14, 16]. In recent years, pasteurization had been re-defined and using high-pressure processing is now considered as a pasteurization methodology [17]. Lactic acid is the most prevalent antimicrobial and processing aid in primary and further processing of meat products in the United States and is one of the main antimicrobials of choice by the meat industry and international regulatory agencies [5, 10, 18, 19].

The purpose of current study was to investigate sensitivity of *E*. *coli* O157: H7 and the six non-O157 serogroups in meat homogenates and ground beef samples exposed to various levels of elevated hydrostatic pressure under controlled temperature. Study additionally compares rifampicin-resistant and wild-type phenotypes as well as sensitivity of ATCC^®^ 43895^™^ (strain involved in the 1992–1993 Pacific Northwest outbreak) to this emerging technology and further investigates role of lactic acid for augmenting pressure-based decontamination of this pathogen of public health concern.

## Materials and methods

### Bacterial strains

For the experiments conducted in ground beef, two strain mixtures (each composed of six strains), and one single strain were utilized separately (total of three inocula). All strains were originally obtained from American Type Culture Collection (Manassas, VA, USA), and were selected based on their epidemiological significance and prior strain selection trials [8, 10]. To facilitate selective enumeration of the inoculated pathogen in presence of background microbiota, immediate rifampicin-resistant variants of strains were prepared in Public Health Microbiology Laboratory by gradual exposure of wild-type strains to rifampicin (Sigma-Aldrich, St. Louis, MO USA) [20]. Previous studies had indicated wild-type and rifampicin-resistant strains of Shiga toxin-producing *E*. *coli* have equivalent sensitivity to antimicrobial interventions and could be used interchangeably in microbiological challenge studies [8, 10]. Current study additionally compared sensitivity of the two phenotypes exposed to hydrostatic pressure and lactic acid as further illustrated earlier.

In addition to presence of background microbiota, as mentioned above, the ground meat samples inoculated separately with six-strain mixture of rifampicin-resistant Shiga toxin-producing *E*. *coli* O157: H7 (STEC), a six-strain mixture of rifampicin-resistant non-O157 (O26, O45, O103, O111, O121, and O145 serogroups) Shiga toxin-producing *E*. *coli* (nSTEC), and a single strain of *E*. *coli* O157: H7 involved in 1992–1993 Pacific Northwest outbreak. The later strain (ATCC^®^ number 43895^™^) was investigated separately due to public health significance of the strain and involvement in a multistate hemorrhagic colitis outbreak [4]. The six strains of rifampicin-resistant STEC were derived from ATCC^®^ strains of BAA 460^™^, 43888^™^, 43894^™^, 35150^™^, 43889^™^, 43890^™^. The nSTEC rifampicin-resistant strains were derived from ATCC^®^ strains of BAA 2196^™^, BAA 2193^™^, BAA 2215^™^, BAA 2440^™^, BAA 2219^™^, BAA 2192^™^. Also known as the “Big Six,” these nSTEC strains belong to the most common non-O157 serogroups associated with human illness in the United States, as further discussed in the introduction [3, 6, 21].

In experiments conducted in meat homogenate, wild-type and rifampicin-resistant six strain mixtures of STEC and nSTEC were used. Strains were identical to the above-mentioned strains of ground meat experiments. Experiments in ground meat were conducted using untreated 80% lean product (80/20 ground meat) in presence of natural background microbiota (mesophilic counts). The experiment conducted in meat homogenate was conducted in 10% sterilized meat homogenate, prepared as described previously [10]. In short, intact fresh beef (top round) was purchased from local market and approximately 2.5 cm of all surfaces were aseptically trimmed and discarded to assure absence of antimicrobial and/or processing aid residue. The 10% (w/w) homogenate was then prepared by homogenizing the fresh beef in distilled water. The homogenate was then aseptically passed through a sterilized cheesecloth to prepare the liquid meat homogenate and sterilized by autoclaving and then cooled at 4°C prior to inoculation, treatments, and microbiological analyses. Absence of background microbiota in the later experiment (experiment conducted in meat homogenate) afforded the opportunity of comparing sensitivity of wild-type and rifampicin-resistant phenotypes of STEC and nSTEC serogroups.

### Bacterial propagation and inoculation

Bacterial strains had been stored as glycerol stock in -80°C freezer. Culturing and inoculum preparation were based on methods explained in our recent study [3]. In short, each strain was individually activated inside 10 mL of Tryptic Soy Broth (Difco, Becton Dickinson, Franklin Lakes, NJ) supplemented with 0.6% of yeast extract (TSB + YE). Addition of yeast extract was based on preliminary trials for minimizing the acid stress of the strains [14]. Activation of the rifampicin-resistant strains were conducted using TSB + YE supplemented with rifampicin (100 μg/mL, Sigma-Aldrich, St. Louis, MO; TSB + Rif). The strains were aerobically incubated at 37°C for 22–24 h. Each strain separately, was sub-cultured by transferring 100 μL of the strain to 10 mL TSB + YE or TSB + Rif for wild-type and rifampicin-resistant strains, respectively. The sub-cultured tubes were then aerobically incubated again at 37°C for 22–24 h. These overnight suspensions (2 mL per strain) were then harvested at 6000 revolutions per min (3,548 g) for 15 min (Centrifuge Model 5424, Eppendorf North America, Hauppauge, NY) to remove growth medium, excreted secondary metabolites, and sloughed cell components. This purification process was repeated by discarding the supernatant and re-suspending the microbial pellets in 2 mL of 1x phosphate-buffered saline (PBS, VWR International, Radnor, PA, USA), using the above-mentioned time and intensity of centrifugation. Supernatant for each strain was then discarded, pellets were re-suspended in PBS and composited to wild-type and/or rifampicin-resistant STEC and nSTEC strain mixtures. As discussed earlier, ATCC^®^ 43895^™^ was prepared as an additional inoculum due to epidemiological and public health significance of the strain. All inocula were then 10-fold serially diluted in PBS for target inoculation level of 6 to 7 log CFU/mL and 6 to 7 log CFU/g, for homogenate and ground meat experiments, respectively.

### High-pressure processing and lactic acid treatments

The ground meat experiment was conducted using 2 grams of inoculated meat inside a no-disk PULSE tube (Pressure BioScience Inc., South Easton, MA USA). The hydrostatic pressure was applied for 0 (untreated control), 1, 3, 5, and 7 min at intensity levels of 650 MPa (94K PSI), 450 MPa (65K PSI), and 250 MPa (36K PSI) using Barocycler Hub880 device (Pressure Bioscience Inc., South Easton, MA, USA). Temperature was controlled at 4°C by a stainless-steel water jacket surrounding the chamber [3, 16]. Water jacket was mechanically linked to a refrigerated circulating water bath (Model refrigerated 1160s, VWR International, Radnor, PA, USA) and temperature values were measured using a type T thermocouple (Omega Engineering Inc., Norwalk, CT, USA) inserted inside the chamber wall and secured with thermal paste (Model 5 AS5-3.5G, Arctic Silver, Visalia, CA, USA). Temperature of 4°C eliminates multiplication of vast majority of meatborne pathogen [10]. Temperature and pressure values were automatically monitored every 3 seconds using Pressure BioScience Inc. Hub Explorer software (V. 1.0.8, PBI, South Easton, MA, USA). Experiments in meat homogenate were similarly conducted inside the PULSE tubes containing 1.5 mL of inoculated homogenate with and without 1% (v/v) lactic acid. The pressure treatments were conducted using Barocycler Hub440 device (Pressure Bioscience Inc., South Easton, MA, USA) for 0 (untreated control), 2, 5, and 7 min at intensity level of 350 MPa (51k PSI) at 4°C using the above-mentioned refrigeration and monitoring procedure. The chamber of Hub440 was inserted with a type K thermocouple (Omega Engineering Inc., Norwalk, CT, USA). The reported exposure time values exclude the time required to reach the pressure (come-up time of 3 seconds for Hub440 unit) and time needed to reach atmospheric pressure after treatment (come-down time of less than 1 second). Our past study had indicated that come-up and come-down times of below one min had negligible (*p* ≥ 0.05) effects on decontamination efficacy of a high-pressure pasteurizer thus microbial reductions observed in the current study could be attributed to effects of processing conditions rather than units’ come-up and come-down times [22]. It is noteworthy that utilization of small sample quantities enables researchers to precisely control temperature, pressure intensity, and exposure time to antimicrobials, yielding results that are generalizable and have external validity. Such precision and accuracy are practically impossible to achieve by larger sample quantities since temperature and pressure would be in fluctuation in various parts of larger experimental conditions.

### Microbiological and pH analyses

The acid exposure of samples in homogenate experiments were precisely controlled by neutralizing the samples immediately after processing and antimicrobial exposure. This was achieved by vortexing (Model Vortex-2 Genie, Scientific Industries, Bohemia, NY, USA) 1000 μL of treated samples with 5 mL of D/E neutralizing broth (Difco, Becton Dickinson, Franklin Lakes, NJ, USA) immediately after treatments. The neutralization allows controlling the exposure time to the lactic acid during the treatments. The pH value of before and after neutralizations were obtained for this experiment using a calibrated pH meter (Mettler Toledo AG, Grelfensee, Switzerland). In ground meat experiment, the 2-gram samples were aseptically transferred to a sterilized filtered bag (Whirl-Pak, Nasco, Modesto, CA) with 10 mL of D/E neutralizing broth and homogenized (200 RPM for 2 min) by a masticator. Homogenized samples were used for microbiological and pH analyses. After treatment and neutralization, samples were 10-fold serially diluted using 1x maximum recovery diluent (MRD, Difco, Becton Dickinson, Franklin Lakes, NJ, USA) to assure recovery of injured but viable cells. Additionally, the non-selective microbiological medium (Tryptic Soy Agar) was supplemented with 0.6% yeast extract to facilitate recovery of pressure-injured cells [15]. During homogenate experiments, wild-type and rifampicin-resistant strain mixtures were spread-plated onto Tryptic Soy Agar (Difco, Becton Dickinson, Franklin Lakes, NJ, USA) supplement with 0.6% yeast extract (TSA + YE) and TSA + YE with 100 μg/mL rifampicin (TSA + Rif), respectively. During the ground meat experiments, conducted with spontaneous rifampicin-resistant pathogens in presence of background microbiota, samples were spread-plated onto TSA +YE (non-selective counts *i*.*e*. mesophilic background microbiota) and onto TSA + Rif (selective counts *i*.*e*. pathogen counts). Plates of both experiments were incubated aerobically at 37°C for 44–48 h for computing colony forming units (CFU).

### Design and statistical analyses

The ground meat and meat homogenate experiments noted above were conducted separately thus the data sets were analyzed separately for each experiment. Each experiment was consisted of two separate biologically independent repetitions (*i*.*e*. two blocks). Each block was further consisted of two replications per time/treatment. Additionally, each replication conducted in two instrumental (microbiological) replications. Thus, in this randomized complete block design, each reported value is mean of 8 independent observations (2 blocks, 2 replications, 2 microbiological repetitions). This selected sample size is based on statistical power analyses conducted and published by Public Health Microbiology program to assure external validity of pressure-based microbiological hurdle validation studies [23]. The microbiological data were log transformed and statistically analyzed using Proc GLM of SAS_9.4_ (SAS Inst., Cary, NC, USA) using an ANOVA procedure followed by Tukey-adjusted (pair-wise comparisons), and Dunnett’s-adjusted (comparing treatments with control) means separation at type I error level of 5%. In GLM procedure, treatment time was considered as the variable in *class* statement. The *model* statement of the procedure was constructed by placing microbial counts = treatment time. *Means* statement included Tukey, and Dunnett code blocks. The inactivation K_max_ and *D*-values was calculated using the best-fitted (maximum R^2^) model obtained by GInaFiT (version 1.7, Katholieke Universiteit, Leuven, Belgium) software [24].

## Results and discussion

### Detection limit, pH, and temperature values

For the study conducted in ground meat, samples were exposed to three levels of elevated hydrostatic pressure (650, 450, and 250 MPa). These samples were treated for 0 min (untreated control) 1, 3, 5, and 7 min, respectively. The pH value of each sample was measured before and after neutralization thus experiments contained control for the treatment and the pH. For the experiments conducted in meat homogenate, samples were treated for 0 (untreated control), 2, 5, and 7 min at 350 MPa with or without presence of 1% lactic acid. The treatment time and intensity levels were selected based on preliminary trials and information from recently published studies to assure selected experimental conditions are in concordance with the practices in the food industry [11, 14–16, 22, 23]. Concentration of lactic acid was selected based on previous literature and relevance for the meat industry application [8, 10].

The pH value is an important intrinsic factor of a product in a microbiological hurdle validation study. For experiments conducted with ground meat, 2 grams of the treated product were neutralized with 10 grams of D/E neutralizing broth prior to stomaching the sample for microbiological analyses. Overall, the pH values of the neutralized samples were 6.99 ± 0.3 (range 5.96 to 7.44; coefficient of variation 4.05%). For the Shiga toxin-producing *E*. *coli* O157: H7 (STEC) and non-O157 Shiga toxin-producing *E*. *coli* (nSTEC) inocula, and the 1992–1993 outbreak strain of the Pacific Northwest, pH values were similar (*p* ≥ 0.05) and were 7.04 ± 0.3 (range 6.04 to 7.44; coefficient of variation 4.60%), 6.99 ± 0.3 (range 5.96 to 7.27; coefficient of variation 3.92%), and 6.92 ± 0.2 (range 5.97 to 7.26; coefficient of variation 3.39%), respectively. For experiments conducted in 10% meat homogenate with 1% (v/v) added lactic acid, pH values were measured before neutralization (after treatment) and after neutralization (before microbiological analyses). For neutralization, 1 mL of sample were exposed to 5 mL of D/E neutralizing broth as noted in materials and methods section. Prior to neutralization, and for samples without added lactic acid, the pH values were similar (*p* ≥ 0.05) and were 5.22 ± 1.1, 5.76 ± 0.7, 5.95 ± 1.0, and 5.84 ± 0.8, for wild-type STEC, wild-type nSTEC, rifampicin-resistant STEC, and rifampicin-resistant nSTEC strain mixtures, respectively. For the same order of strain mixtures and for samples containing 1% (v/v) lactic acid prior to neutralization, the pH values were also not different (*p* ≥ 0.05) and were 2.52 ± 0.5, 2.44 ± 0.2, 2.47 ± 0.2, 2.59 ± 0.2, respectively. For same order of stain mixtures and after neutralization, pH values (*p* ≥ 0.05) for homogenate samples without added lactic acid were 7.05 ± 0.3, 7.14 ± 0.1, 7.12 ± 0.4, 7.23 ± 0.3, respectively. For homogenate acidified with 1% lactic acid (v/v) and after 1 to 5 neutralization with D/E neutralizing broth, pH values of wild-type STEC, wild-type nSTEC, rifampicin-resistant STEC, and rifampicin-resistant nSTEC strain mixtures were similar (*p* ≥ 0.05) and were 6.10 ±0.2, 5.87 ± 0.2, 6.23 ± 0.3, and 6.11 ± 0.4, respectively.

Detection limit of the experiment in 10% meat homogenate was 0.78 log CFU/mL, and the experiments were conducted at target temperature of 4°C. Before treatment, temperature was 3.9 ± 0.2 (range 3.2 to 4.3; coefficient of variation 5.7%). The temperature remained constant (*p* ≥ 0.05) after the treatment and was 3.9 ± 0.3 (range 3.1 to 4.4; coefficient of variation 7.1%). Detection limit of ground meat experiments was 1.08 log CFU/g and trials were similarly conducted under the controlled temperature of 4°C.

### Decontamination of ground meat at 250 to 650 MPa

These experiments were conducted by inoculating ground meat with rifampicin-resistant inocula and exposing the products containing background microbiota to three levels of elevated hydrostatic pressure at 250, 450, and 650 MPa. Thus, counts of selective medium (TSA + Rif) represents colony forming units (CFU) of the rifampicin-resistant pathogen while counts of non-selective medium (TSA +YE) represents counts of mesophilic background microbiota. In addition to the two strain cocktails of STEC and nSTEC, ATCC^®^ 43895^™^ was additionally used as the third inoculum, due epidemiological significance of the strain, as further elaborated in previous section. ATCC^®^ 43895^™^ as well belongs to O157 serogroup of *E*. *coli* [25].

Pressure intensity level of 250 MPa is considered a low level of pressure in food processing. Yet this intensity is nearly two times higher than highest pressure observed in Mariana Trench, the deepest oceanic trench on earth [26].

**Fig 1:**
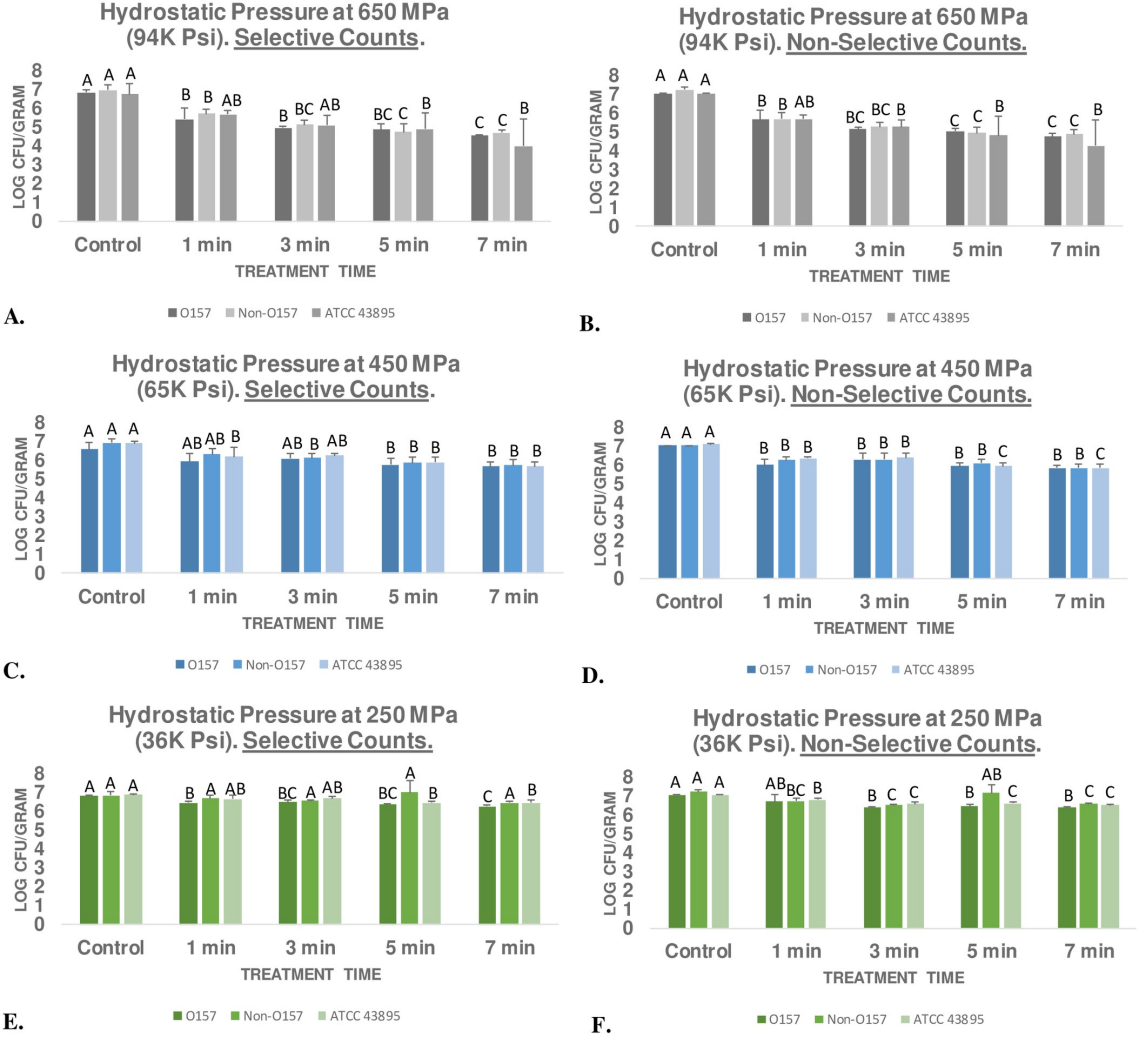
Ground meat experiment for evaluating effects of elevated hydrostatic pressure against background microbiota and six-strain mixture of rifampicin-resistant *E*. *coli* 0157:H7, the ‘Big Six’ rifampicin-resistant non-0157 *E*. *coli* six-strain mixtures, and the 1991–1992 Pacific Northwest outbreak rifampicin-resistant strain. Samples treated (Barocycler Hub880, Pressure BioScience Inc., South Easton, MA) at 4°C using selective (TSA + Rif) and non-selective (TSA + YE) medium (mesophilic bacterial counts). Within each graph, columns of each treatment time followed by different uppercase letters are representing log CFU/g values that are statistically *(p* < 0.05) different (Tukey-adjusted ANOVA). **A.** Hydrostatic pressure at 650 MPa- selective counts; **B.** Hydrostatic pressure at 650 MPa- mesophilic bacterial counts; **C.** Hydrostatic pressure at 450 MPa- selective counts; **D.** Hydrostatic pressure at 450 MPa- mesophilic bacterial counts; **E.** Hydrostatic pressure at 250 MPa- selective counts; **F.** Hydrostatic pressure at 250 MPa- mesophilic bacterial counts. Overall pH of neutralized samples = 6.99 ± 0.3 (range 5.96 to 7.44; coefficient of variation = 4.05%).

At this low intensity and at 4°C, even after 7 min of treatment no (*p* ≥ 0.05) or negligible (*i*.*e*. less than one log) reductions were observed- selective counts of STEC, nSTEC, and ATCC^®^ 43895^™^ were reduced by only 0.56, 0.38, and 0.41 log CFU/g, respectively (Fig 1E). Corresponding mesophilic bacterial count reductions were similarly not significant or had low biological significance and were 0.68, 0.64 and 0.52 log CFU/g, respectively (Fig 1F). Mild hydrostatic pressure at 450 MPa and at 4°C resulted in appreciable (*p* < 0.05) reductions in pathogen and mesophilic background microbiota counts for treatments longer than one min (Fig 1C and 1D). After three min of treatment, the time duration most common in food industry for high-pressure processing [14, 16], selective counts of the STEC, nSTEC, and ATCC^®^ 43895^™^ were reduced by 0.48, 0.80, and 0.65 log CFU/g, respectively (Fig 1C). At 7 min, the pathogen reductions observed were 0.90, 1.19, and 1.21 log CFU/g, respectively (Fig 1C).

Similarly, after 7 min of treatment, mesophilic bacterial counts of the samples were reduced by 1.21, 1.21, and 1.27 log CFU/g, respectively (Fig 1D). Predictably, treatment at 650 MPa was capable of more enhanced reductions of the pathogen and mesophilic background microbiota counts (Fig 1A and 1B). Prior to treatment at 650 MPa, selective counts of STEC, nSTEC, and ATCC^®^ 43895^™^ were 6.86 ± 0.2, 6.98 ± 0.3, and 6.77 ± 0.5 log CFU/g (Fig 1A). After three min of treatment at 650 MPa (at 4°C), these counts were reduced (*p* < 0.05) to 4.99 ± 0.1, 5.13 ± 0.2, and 5.6 ± 0.5 log CFU/g and after 7 min of treatment these counts were reduced (*p* < 0.05) to 2.30, 2.26, and 2.75 log CFU/g (Fig 1A). This indicates that treatment at 650 MPa for 7 min could eliminate up to >99% of the pathogen. These reductions could be very desirable for the meat industry, currently lactic acid treatment, one of the main antimicrobial intervention in primary processing of meat products, even at high temperature of 55°C, offer 1.2 to 1.5 log CFU/mL reduction of O157 and non-O157 *E*. *coli* on beef trimming [8, 18].

Results of these experiments indicate that non-O157 serogroups have very similar sensitivity to hydrostatic pressure and lactic acid compared to *E*. *coli* O157:H7. Thus, a process validated for decontamination of *E*. *coli* O157:H7, could almost certainly reduce the non-O157 serogroups as well. Additionally, the 1992–1993 outbreak strain used in this study (ATCC^®^ 43895^™^) was reduced in a rate comparable to *E*. *coli* O157:H7 and non-O157 serogroups indicating that this epidemiologically important strain did not have any unusual resistance to elevated hydrostatic pressure and lactic acid. Calculation of inactivation indices revealed similar trends (Table 1). The *D*-values, amount of time (min) required for 90% reduction of the pathogen inoculated in ground meat and exposed to elevated hydrostatic pressure of 650, 450, and 250 MPa at 4°C were 2.97, 7.00, and 18.98 min for ATCC^®^ 43895^™^, respectively (Table 1). The corresponding reductions for STEC strain-mixture were 3.71, 9.60, and 16.00 min, respectively (Table 1). K_max_ inactivation indices (1/min) calculated based on best-fitted non-linear model exhibited similar trends comparing the three inocula (Table 1). Results of our experiment are in concordance with previous literature were treatment of ground meat samples at 350 MPa for 9 min at 4°C resulted in 0.7 to 1.3 log CFU/g of *E*. *coli* O157: H7, with higher reductions were also reported at 600 MPa [27]. Other researchers also observed 1.57 to 3.49 log CFU/g reduction of *E*. *coli* O157: H7 after 3-min treatment at 400 MPa [28]. Using a nonpathogenic surrogate for *E*. *coli* O157: H7 and non-O157 serogroups, there had also been report of 0.9 to 1.8 log CFU/g reductions at 200 MPa, 2.5 to 3.6 log CFU/g reductions at 400 MPa, 4.5 to 5.6 log CFU/g reductions at 600 MPa [29]. The results of the later study exhibit unusually higher log reductions than this study and previously reported studies. The deviation of this particular study with existing literature could be attributed to lack of utilization of pathogenic organisms (*i*.*e*. use of a surrogate) in their study and/or inadequate control of processing temperature.

**Table 1:** From the ground meat experiment, inactivation indices of six-strain cocktail of rifampicin-resistant phenotype of *E*. *coli* O157:H7, the ‘Big Six’ non-O157 *E*.*coli* strain mixtures, and the 1991–1992 Pacific Northwest outbreak rifampicin-resistant strain exposed to elevated hydrostatic pressure.

Pathogen	Treatement	*D*-value^a^	K_max1_^b^	K_max2_	R^2^
***E*. *coli* O157:H7**	650 MPa	3.71	4.20	0.25	0.90
	450 MPa	9.60	27.98	0.13	0.50
	250 MPa	16.00	25.41	0.08	0.86
**Non-O157 *E*. *coli***	650 MPa	3.47	3.31	0.23	0.91
	450 MPa	6.61	3.56	0.23	0.74
	250 MPa	19.76	0.05	0.05	0.60
**ATCC**^**®**^ **43895**^**™**^	650 MPa	2.97	23.69	0.59	0.60
	450 MPa	7.00	27.31	0.21	0.68
	250 MPa	18.98	25.01	0.09	0.59

^a^ *D*-value (min) was calculated as the reciprocal of the positive slope of the best-fitted model (goodness-of-fit indicator of R^2^ values, α = 0.05), resulting from plotting of the pathogen counts (log CFU/mL) as affected by treatments.

^b^ K_max_ values (1/min) are selected using the GInaFiT software. K_max_ values indicate the expressions of number of log cycles of reduction in 1/min unit.

### Sensitivity of wild-type Shiga toxin-producing *E*. *coli* in meat homogenate

**Fig 2:**
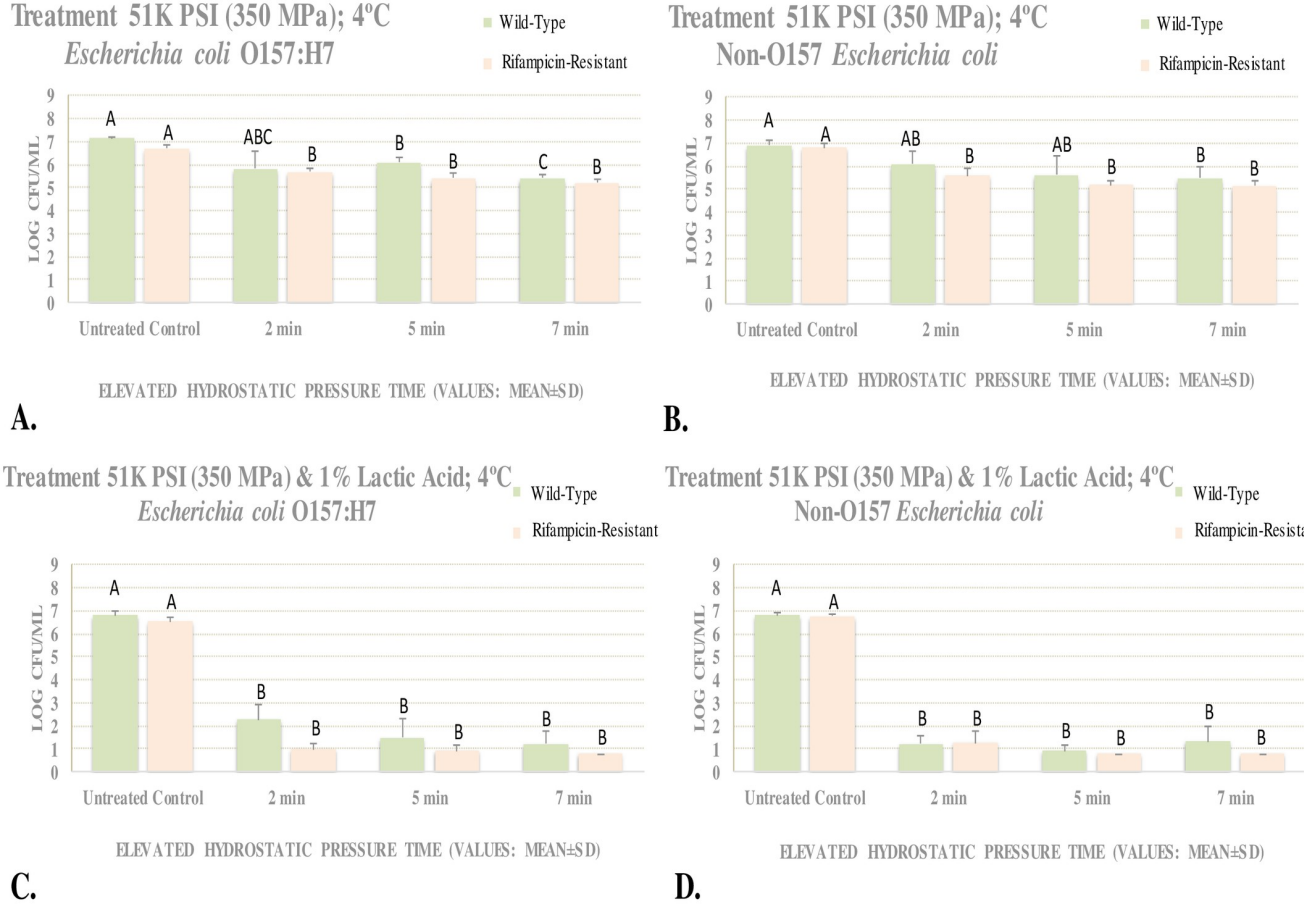
Meat homogenate experiment for evaluating effects of elevated hydrostatic pressure against six-strain cocktail of wild-type and rifampicin-resistant phenotypes of *E*. *coli* 0157:H7 and the ‘Big Six’ non-0157 *E*. *coli* six-strain mixtures. Samples treated at 350 MPa (Barocycler Hub440 Explorer, Pressure Bioscience Inc., South Easton, MA, USA), with and without 1% lactic acid, exposed for 0, 2, 5, and 7 min at 4°C. Within each graph, columns of each treatment time followed by different uppercase letters are representing log CFU/mL values that are statistically *(p* < 0.05) different (Tukey-adjusted ANOVA). **A.** *E*. *coli* 0157 counts without lactic acid; **B.** *E*. *coli* 0157 counts with lactic acid; **C.** Non-0157 *E*. *coli* counts without lactic acid; **D.** Non- *E*. *coli* 0157 counts with lactic acid. The pH of 10% meat homogenate = 6.11 ± 0.1; the pH of acidified homogenate = 2.52 ± 0.5 and 2.44 ± 0.2 (before neutralization) and 5.22 ± 1.1 and 5.76 ± 0.7 (after neutralization) for wild-type 0157 and non-0157serogoups, respectively.

These experiments examined the efficacy of elevated hydrostatic pressure at 350 MPa for reduction of O157 (STEC) and non-O157 Shiga toxin-producing *E*. *coli* (nSTEC) in meat homogenate. The effects of 1% (v/v) lactic acid were additionally investigated, and sensitivity of wild-type and rifampicin-resistant strains were compared. These experiments were conducted in absence of background microbiota in 10% sterilized meat homogenate. At this medium level of elevated hydrostatic pressure (350 MPa), 1.7 and 1.4 log CFU/mL reductions (*e*.*g*. between 90–99%) were observed for wild-type STEC and nSTEC serogroups, respectively, after 7 min of treatment at 4°C (Fig 2A and 2B). Presence of lactic acid, to a great extent augmented (*p* < 0.05) the efficacy of this treatment. At 350 MPa (at 4°C), and after 7 min, the reductions of wild-type STEC and nSTEC serogroups were similar with each other (*p* ≥ 0.05) and were 5.6 and 5.5 (*e*.*g*. >99.999%) log CFU/mL (Fig 2C and 2D). It is important to note that, as further detailed in materials and methods section, this study was conducted at 4°C under precise temperature control and monitoring, thus, reductions observed in current trials could be attributed solely to pressure and/or lactic acid interventions rather than temperature fluctuations during treatment.

Reductions at lower treatment times in presence and absence of lactic acid were similar to reductions obtained at 7 min (Fig 2A–2D). Counts of wild-type STEC and nSTEC were similar after exposure to vast majority of time/pressure/lactic acid interventions. As further elaborated in the introduction, *E*. *coli* O157: H7 had an important role in meat interstate regulatory affairs since the 1990s and thus this pathogen is the primary serogroup used in literature for relevant hurdle validation studies [5, 19]. Non-O157 serogroups of the pathogen have received regulatory and industry attention only in recent years and limited microbiological challenge studies are available comparing non-O157 serogroups to *E*. *coli* O157: H7 [3, 8].

Our study illustrates that both O157 and non-O157 serogroups have similar sensitivity to the high-pressure and lactic acid treatments. This similarity thus shows that if a commercial decontamination program is validated against *E*. *coli* O157: H7, it is almost certainly capable of eliminating non-O157 serogroups of the pathogen as well.

Our study additionally showcase the efficacy of lactic acid for augmenting pressure-based decontamination of this pathogen that could be of great importance to the meat industry since lactic acid is the most widely used antimicrobial in peri- and post-harvest processing of meat products in North America [8, 10, 18]. Our study indicated even a mild pressure treatment in presence of 1% lactic acid could lead to reduction of >5 logs of the wild-type pathogen. This could be of great importance for improving the safety of moisture-enhanced and non-intact meat products, beef trimmings, and ground meat, that are the vast majority of beef carcass fabrication cuts in the market [8, 19].

It is important to note that in addition to the meat industry that utilizes lactic acid as a processing aid and/or antimicrobial agent, this compound had been part of various regulatory agencies reports and recommendations. A joint task force of Food and Agriculture Organization and the World Health Organization, as an example, had reported on utilization of lactic acid for minimizing the risk of foodborne pathogens of public health concern in meat industry [18].

**Fig 3:**
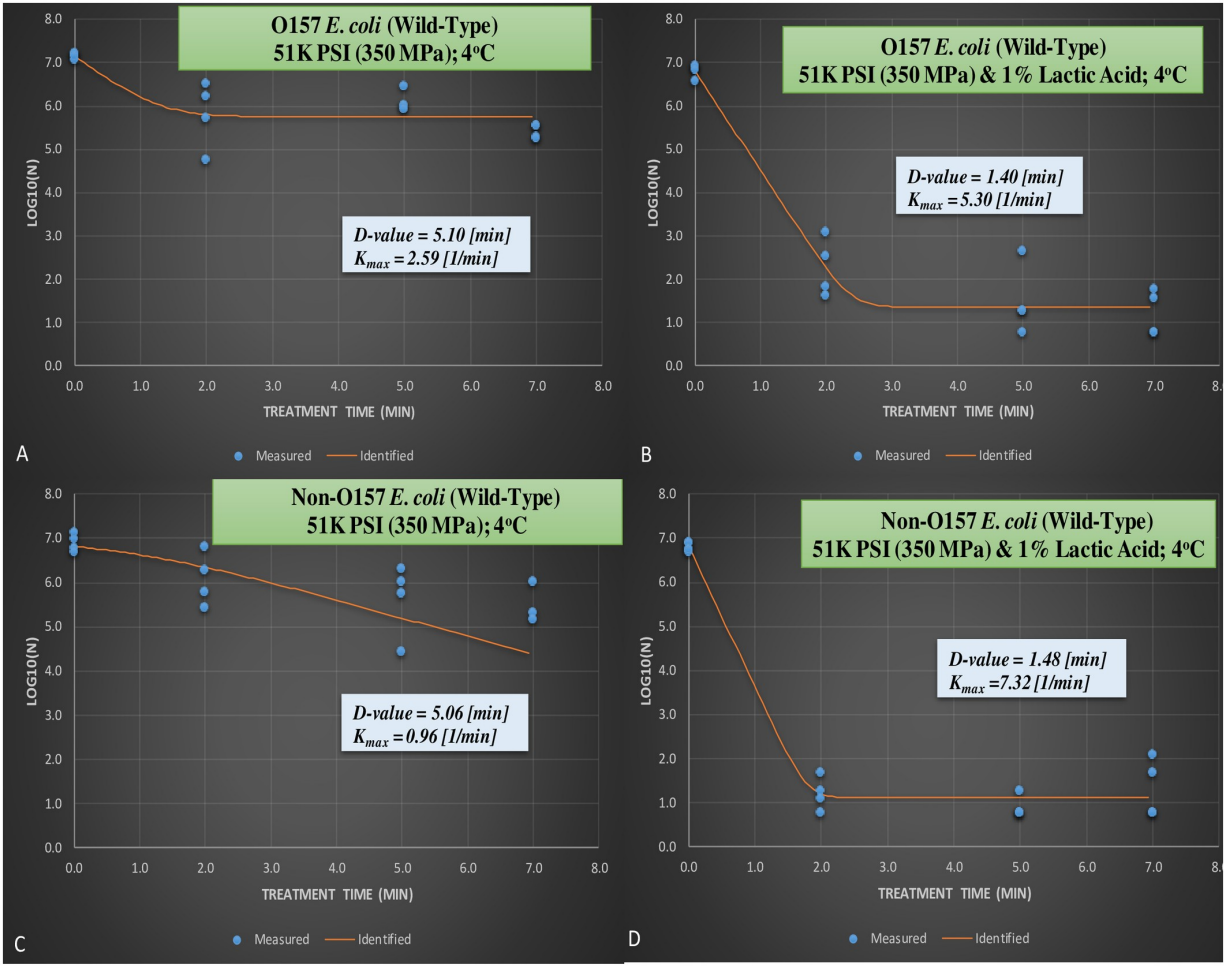
From the meat homogenate experiment, inactivation of six-strain cocktail of wild-type phenotype of *E*. *coli* 0157:H7 and the ‘Big Six’ non-0157 *E*.*coli* strain mixtures treated at elevated hydrostatic pressure at 350 MPa and 1% lactic acid (Barocycler Hub440 Explorer, Pressure Bioscience Inc., South Easton, MA, USA) for 0, 2, 5, and 7 min at 4°C. **A.** Wild-type *E*. *coli* 0157 at 350 MPa without lactic acid; **B.** Wild-type *E*. *coli* 0157 at 350 MPa with 1% lactic acid; **C.** Wild-type non-0157 *E*. *coli* at 350 MPa without lactic acid; **D.** Wild-type non-0157 *E*. *coli* at 350 MPa with 1% lactic acid.

**Fig 4:**
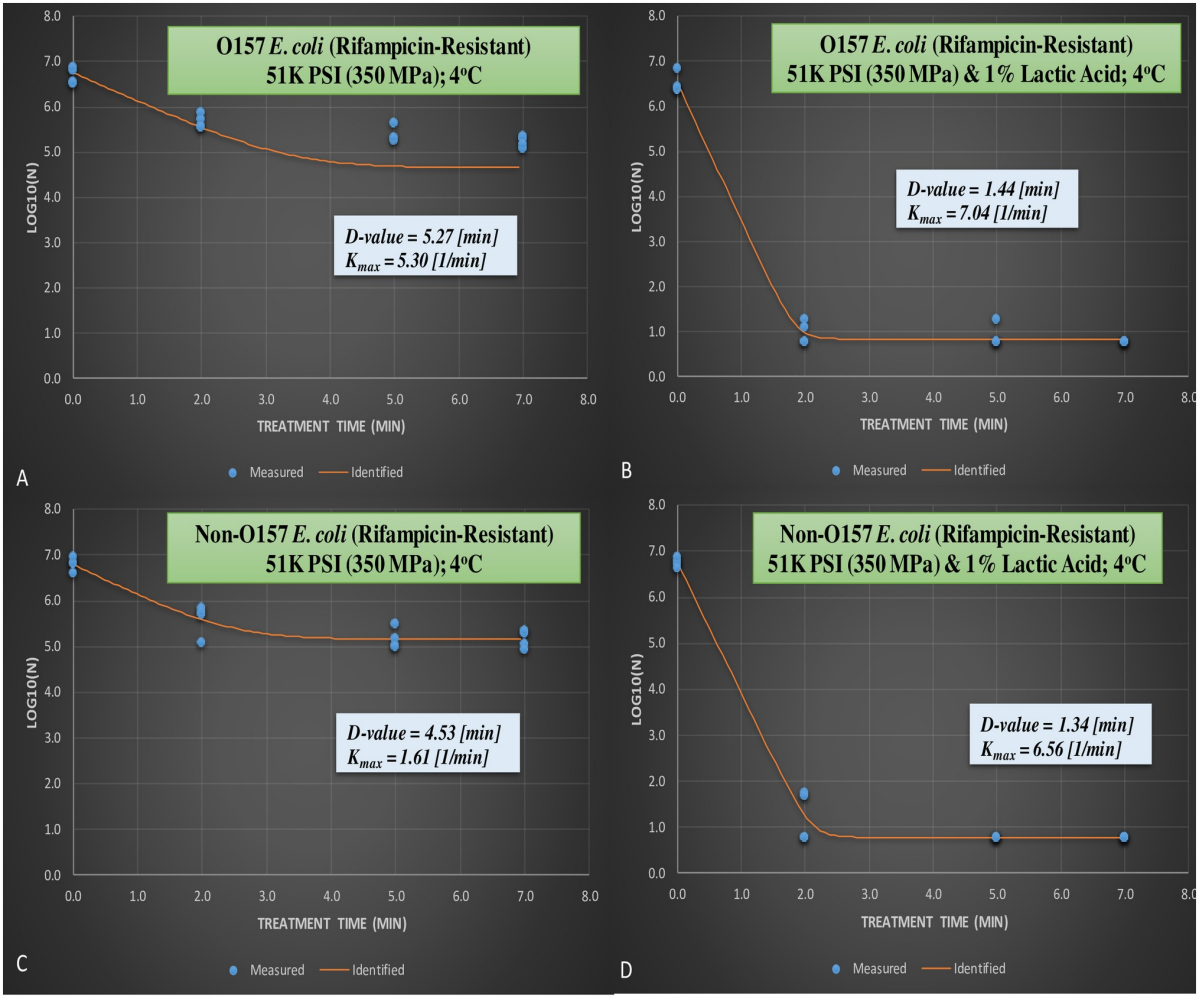
From the meat homogenate experiment, inactivation of six-strain cocktail of rifampicin-resistant phenotype of *E*. *coli* 0157:H7 and the ‘Big Six’ non-0157 *E*.*coli* strain mixtures treated at elevated hydrostatic pressure at 350 MPa and 1% lactic acid (Barocycler Hub440 Explorer, Pressure Bioscience Inc., South Easton, MA, USA) for 0, 2, 5, and 7 min at 4°C. **A.** Rifampicin-resistant *E*. *coli* 0157 at 350 MPa without lactic acid; **B.** Rifampicin-resistant *E*. *coli* 0157 at 350 MPa with 1% lactic acid; **C.** Rifampicin-resistant non-0157 *E*. *coli* at 350 MPa without lactic acid; **D.** Rifampicin-resistant non-0157 *E*. *coli* at 350 MPa with 1% lactic acid.

The reductions observed for wild-type serogroups and efficacy of lactic acid for augmenting pressure-based decontamination of the pathogen were additionally exhibited by calculation of inactivation indices. *D*-values, amount of time (in min) needed for 90% reduction of the wild-type pathogen inoculated in 10% meat homogenate and exposed to 350 MPa hydrostatic pressure at 4°C were 5.10 and 5.06 min for STEC and nSTEC serogroups, respectively, in absence of lactic acids (Figs 3A and 4C). Moreover, the presence of 1% lactic acid reduced the *D*-values for the aforesaid treatment groups to 1.40 and 1.48 min, respectively (Figs 3B and 4D). Results obtained from best fitted non-linear model as expressed by K_max_ (1/min) also exhibits similar trends (Figs 3A and 4D).

### Sensitivity of rifampicin-resistant Shiga toxin-producing *E*. *coli* in meat homogenate

The rifampicin-resistant variants of Shiga toxin-producing *E*. *coli* are of particular importance in validation studies. Development of resistance to a medically important antibiotic such as rifampicin is of public health importance and is of interest to assimilate whether a treatment capable of eliminating wild-type pathogen is also efficacious for removal of the rifampicin-resistant variants [30]. Resistance to one antibiotic could as well be an indicator for presence of other antibiotic resistance genes [31, 32]. From a microbiology perspective, utilization of rifampicin-resistant phenotype of Shiga toxin-producing *E*. *coli* is also of great importance. The *E*. *coli* O157: H7 does own a selective and differential medium (Sorbitol-MacConkey Agar) that allows researcher to isolate the bacterium using culture-based methods and more importantly using the selective medium for differentiating between the inoculated pathogen and existing background microbiota of a product [3, 33]. However, the non-O157 serogroups of *E*. *coli* currently do not have a validated selective and differential medium for each serogroup, thus researchers have difficulty conducting inoculation-based microbiological challenge studies in presence of background microbiota for non-O157 serogroups. Utilizing the rifampicin-resistant strains of Shiga toxin-producing *E*. *coli*, researchers could differentiate between the natural microbiota of a product and the inoculated rifampicin-resistant pathogen by adding rifampicin to non-selective media and thus reporting selective (representing rifampicin-resistant inoculum) and non-selective (representing background microbiota) counts that enables examining decontamination efficacy of a treatment in presence of natural microbiota [8, 11, 34].

Under the condition of our experiment, at 350 MPa and at 4°C, counts of rifampicin-resistant STEC and nSTEC were 6.68 ± 0.2 and 6.80 ± 0.2 log CFU/mL, respectively (Fig 2A and 2B). Counts for the above-mentioned order of phenotypes were reduced (*p* < 0.05) to 5.40 ± 0.2 and 5.17 ± 0.2 log CFU/mL after 5 min of treatment and were reduced to (*p* < 0.05) to 5.23 ± 0.1 and 5.15 ± 0.2 log CFU/mL after 7 min of treatment at 350 MPa at 4°C, respectively (Fig 2A and 2B). Thus, STEC and nSTEC exhibited modest reductions of up to 1.5 and 1.6 when exposed to 350 MPa of hydrostatic pressure at 4°C, respectively. Addition of 1% lactic acid to great extent augmented (*p* < 0.05) the decontamination efficacy of this treatment. Counts of rifampicin-resistant STEC on time 0 (before treatment) and after 2, 5, and 7 min of exposure to 350 MPa treatment (at 4°C) in presence of 1% lactic acid were 6.51 ± 0.2, 0.97 ± 0.2, <0.90 ± 0.2, and <0.78 ± 0.0 log CFU/mL, respectively (Fig 2C). Similarly, counts of rifampicin-resistant STEC for the above-mentioned order of treatment times were 6.75 ± 0.1, <1.24 ± 0.5, <0.78 ± 0.0, and <0.78 ± 0.0, respectively (Fig 2D). Thus, STEC and nSTEC exhibited reductions of up to 5.7 and 6.0 log CFU/mL when exposed to 350 MPa hydrostatic pressure in presence of 1% lactic acid, respectively.

Overall, log reductions of wild-type and rifampicin-resistant phenotypes and STEC and nSTEC serogroups were similar. As an example, after 7 min of exposure to elevated hydrostatic pressure at 4°C, log reductions of wild-type STEC, wild-type nSTEC, rifampicin-resistant STEC, and rifampicin-resistant nSTEC were 1.7, 1.4, 1.5, and 1.6 CFU/mL, respectively (Fig 2A and 2B). For same order of pathogen phenotypes/serogroups and same intensity level of treatment and in presence of 1% lactic acid, these reductions were 5.6, 5.5, 5.7, and 6.0 log CFU/mL, respectively (Fig 2C and 2D).

These findings are in concordance with existing literature, where treatments of 300, 350 and 400 MPa were augmented with addition of 1% acidic additives and resulted in up to 6-log reductions of various serogroups of Shiga toxin-producing *E*. *coli* [35]. Similar results had been reported using hydrostatic pressure at 350 MPa for achieving up to 5 log reductions of *E*. *coli* O157: H7 [36].

Since wild-type and rifampicin-resistant pathogens exhibited comparable (*p* ≥ 0.05) sensitivity to hydrostatic pressure and hydrostatic pressure and lactic acid, it is concluded that these two phenotypes could be used interchangeably in microbiological hurdle validation studies. Similar conclusions had been reported previously [10].

Linear and non-linear inactivation indices obtained in current study additionally confirms these similarities and efficacy of lactic acid to augment the decontamination efficacy of pressure-based treatments (Fig 4A–4D). The *D*-value and K_max_ value for rifampicin-resistant STEC were 5.27 (min) and 5.30 (1/min), respectively (Fig 4A) for samples treated inside 10% meat homogenate at 350 MPa, at 4°C, and for up to 7 min. Lactic acid to great extent increased the efficacy of the treatment, corresponding values for STEC and nSTEC in presence of lactic acid were 1.44 (min) and 7.04 (1/min) and 1.34 (min) and 6.56 (1/min), respectively (Fig 4B and 4D). This indicates that, with linearity assumption, 5.27 min of this treatment is needed to reduce 90% of rifampicin-resistant STEC without lactic acid. Presence of lactic acid reduces time needed for 90% reduction of the pathogen to 1.44 min.

It is important to mention that lactic acid is one of the most commonly used antimicrobials in primary processing of the red meat in the United States. However, use of lactic acid is primarily in form of processing aid where the antimicrobial is applied on surface of potentially contamination products and then rinsed by subsequent treatments. This study explored addition of lactic acid as an antimicrobial incorporated in product formulation to assure enhanced safety of the product at the processing step and during shelf-life. Although no visible negative organoleptic characteristics of product with 1% lactic acid was observed during the current study, prior to adopting this practice, more in-depth sensory analyses of the product with 1% lactic acid during the shelf life is recommended. Our results indicate that lactic acid as an ingredient has a great potential to enhance safety of non-intact meat products.

## Conclusions

Under the condition of our experiments conducted in meat homogenate, it has been observed that wild-type and rifampicin-resistant phenotypes of *E*. *coli* O157: H7 and non-O157 serogroups have comparable sensitivity to elevated hydrostatic pressure. Thus rifampicin-resistant and wild-type phenotypes could be used interchangeably in microbiological hurdle validation studies. Elevated hydrostatic pressure of 350 MPa reduced the wild-type *E*. *coli* O157: H7 for 1.1 to 1.3 log CFU/mL. Wild-type non-O157 serogroups exhibited comparable sensitivity to pressure and were reduced for 0.8 to 1.4 log CFU/mL. Presence of 1% lactic acid to great extent augmented the decontamination efficacy of pressure-based treatments leading to 5.6, 5.5, 5.7, and 6.0 log CFU/mL reductions of wild-type *E*. *coli* O157: H7, wild-type non-O157 serogroups, rifampicin-resistant *E*. *coli* O157: H7, and rifampicin-resistant non-O157 serogroups, respectively.

Under conditions of our experiments conducted in ground meat, elevated hydrostatic pressure at 250 MPa had no or modest (<1 log CFU/g) decontamination efficacy against rifampicin-resistant *E*. *coli* O157: H7, rifampicin-resistant non-O157, and ATCC^®^ 43895^™^ strain. Treatment of 650 MPa were efficacious for 1.26 to 2.75 log CFU/g reductions of the tested phenotypes/strain. The O157 and non-O157 serogroups exhibited similar sensitivity to elevated hydrostatic pressure indicating that a process validated against *E*. *coli* O157: H7, is almost certainly capable of eliminating vast majority of non-O157 serogroups as well.

## Supporting information

S1 File (XLSX)
